# Supplementary material for: Co-Expression of Coxsackievirus/Adenovirus Receptors and Desmoglein 2 in Lung Adenocarcinoma: A Comprehensive Analysis of Bioinformatics and Tissue Microarrays
Source: J Clin Med. 2020 Nov 18;9(11):3693. doi: 10.3390/jcm9113693 (PMC7698609; doi:10.3390/jcm9113693)
Supplement: Supplementary file 1 [file jcm-09-03693-s001.pdf]

Sup. Table 1. List of GEO data sets

GEO Accession number	# of sample	Pubmed ID	downloaded data
**GSE102511**	48	28951454	GSE102511_Smruthy_etal_allsamples_TPM.txt.gz
**GSE52248**	18	24735754	GSE52248_FPKM_genes.txt.gz
**GSE68571**	77	12118244	GSE68571_series_matrix.txt.gz
**GSE32863**	30	22613842	GSE32863_series_matrix.txt.gz

Sup. Table 2. Gene names

KRT8	XRCC2	CHMP4C	PTPRQ	TGFBR3	STK4	GATA2
LIMK1	LAD1	LRIG3	ANGPT1	TJP1	SRPX	PCDH10
EPCAM	LCN2	SERINC2	MICA	WNT2	NEDD9	WNT3A
MDM2	S100A11	SEMA4B	PTH1R	ZFP36	PDPN	HYAL1
MKI67	KIF20A	PVT1	AQP1	HYAL2	MCAM	COL4A6
MMP7	SMAD1	ADRB2	RUNX1T1	SOCS2	FOSB	DACH1
MMP13	MCM4	FAS	BCL2L2	WISP2	MARCO	MXI1
IRF4	MMP11	CDKN1C	EDNRB	SOCS3	DNAJB4	PLCD3
MYBL2	PTK6	GJA1	BMPR2	EFNB2	MME	TLR4
CEACAM6	SPRR1A	GLI3	CAV2	GZMB	DMBT1	MSR1
NT5E	CD28	JAG1	KLF6	SEMA3F	PRDM2	PRKCDBP
OCLN	IRF6	TIMP3	CST6	KLF4	HEY1	PMP22
PAK1	LGALS4	TTPA	EPAS1	BMPR1A	STAT5B	RASSF2
PDK1	POU2AF1	PECAM1	CYR61	DMPK	PPARGC1	IL16
PTK7	CCL19	ELN	CXCR2	DUSP1	A	NFATC1
RAD51	VARS	LDLR	CAV1	ETV5	PPP1R15A	PPFIBP1
MRPL12	CXCL13	TAP2	CEACAM3	FGF10	LATS2	STARD13
CCL22	TCEA1	FCGR3A	CTGF	NOTCH4	ADARB1	NCAM1
SDC1	PIM2	IL15	HBEGF	PARK2	STK4	ITGA1
SOX4	PMEL	TNF	EGR1	VIPR1	SRPX	GRASP
SPINK1	UBE2C	NOS3	FGF2	WNT7A	NEDD9	ERG
SPRR1B	OIP5	CXCR1	FOXO1	SEMA3B	PDPN	FLT4
TFAP2A	CBLC	FASLG	FLI1	SLIT2	MCAM	TSC22D1
TFAP2C	BLNK	ADH1B	FLT1	KL	FOSB	NEDD4
TIMP1	DDR1	ALDH1A1	GNA11	CDKN2B	MARCO	RAPGEF1
TK1	IKBKE	CSF1	GNAI2	ROR1	DNAJB4	GAB1
VDAC1	B3GNT3	HPGD	GPX3	PRF1	MME	FGFR4
ADAM12	RHOD	KCNJ5	CXCL2	FEZ1	DMBT1	HMGA1
TNFRSF25	DLGAP5	PTGS2	HOXA4	CALM3	PRDM2	
MPZL1	ARHGAP1	GATA3	HOXB5	ETS2	HEY1	

PRC1	1A	ANXA2	ID1	FGR	STAT5B
ATIC	MELK	NUMB	IL7R	FOS	PPARGC1
PTTG1	LEF1	FAT3	ITGA5	GATA6	A
RECQL4	SLC35F2	NTRK3	ITGAL	GFRA1	PPP1R15A
BUB1	LAPTM4B	ZBTB16	JUNB	JUND	LATS2
CDH1	ANLN	NR3C1	KDR	PRKCE	ADARB1
CEACAM5	EML4	MYADM	LEPR	VEGFC	STK4
COX6C	LARS	CD34	MEIS1	AKT3	SRPX
EZH2	PMAIP1	SSTR1	MYH9	ABCA1	NEDD9
LRP8	FANCE	ACVRL1	PBX1	HLA-E	PDPN
CLTC	MS4A1	ITGA6	PDGFA	HSD11B1	MCAM
GDF15	FOXM1	MYO1C	PDGFB	LYL1	FOSB
CXCL14	ST14	PXN	HHEX	SMAD6	MARCO
AQP3	PERP	PLAGL1	MAPK3	PYGM	DNAJB4
MMP9	PLA2G4A	TCF4	PTPRC	LHFP	MME
SLC4A3	DSC2	MCC	PTPRD	FSTL3	DMBT1
TNFSF15	CCNB1	SEPP1	S100A4	SMAD7	PRDM2
CTLA4	FAM83A	FLNA	S100A8	MLLT1	HEY1
NR2F6	FANCD2	NACA	CCL3	TBX2	STAT5B
F2RL1	TP53INP1	CD36	CCL4	DDR2	PPARGC1
GRB7	DLX4	TGFB2	CCL5	PDGFRA	A
HINT1	MSI2	SGK1	TAL1	RRAS	PPP1R15A
					LATS2
					ADARB1D
					AB2IP

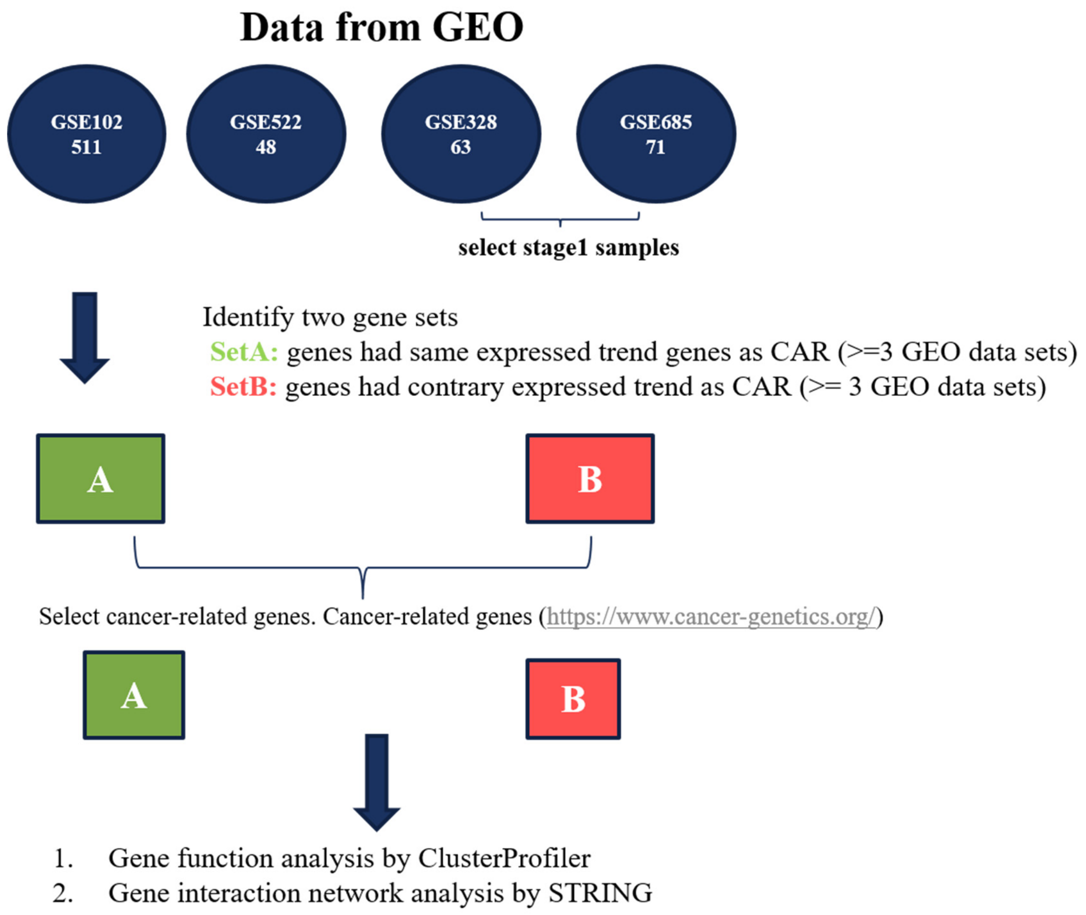

Sup. Figure S1. Analysis flow of identification two groups of genes on CAR sequential changes from NL, atypical adenomatous hyperplasia (AAH) to invasive LUAD. Two groups of genes were also used to do gene function and gene interaction network analysis.

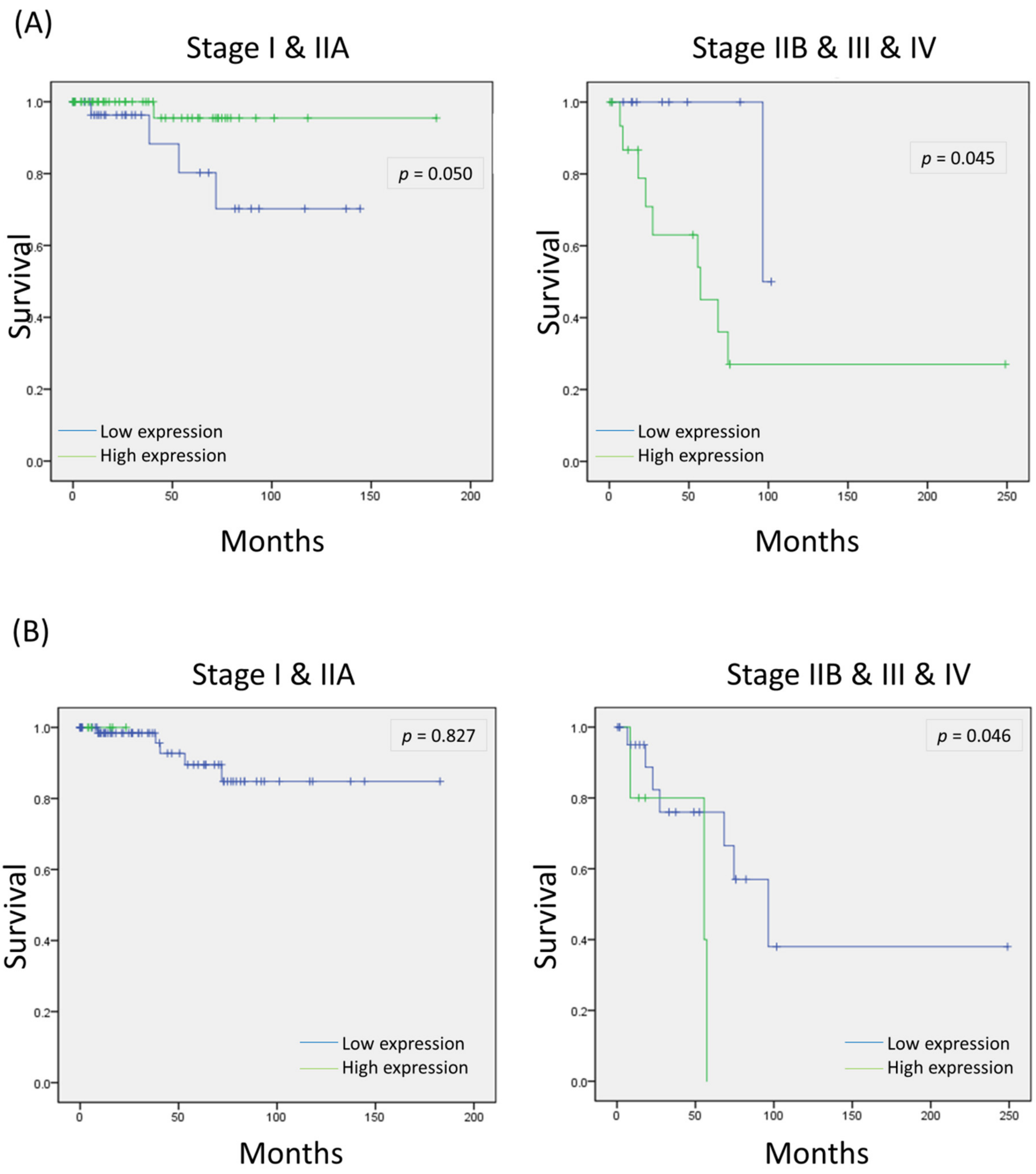

Sup. Figure S2. The impacts of expression level of (A) CAR and (B) DSG2 on survival in early stage disease (Stage I and IIA) and late stage disease (Stage IIB and III and IV).
